# Cool and menthol receptor TRPM8 in human urinary bladder disorders and clinical correlations

**DOI:** 10.1186/1471-2490-6-6

**Published:** 2006-03-06

**Authors:** Gaurav Mukerji, Yiangos Yiangou, Stacey L Corcoran, Inger S Selmer, Graham D Smith, Christopher D Benham, Chas Bountra, Sanjiv K Agarwal, Praveen Anand

**Affiliations:** 1 Peripheral Neuropathy Unit, Hammersmith Hospital and Imperial College London, UK; 2 Department of Urology, Hammersmith Hospital and Imperial College London, UK; 3 Neurology and GI CEDD, GlaxoSmithKline Research and Development Ltd, New Frontiers Science Park (North), Harlow, Essex, CM19 5AW, UK

## Abstract

**Background:**

The recent identification of the cold-menthol sensory receptor (TRPM8; CMR1), provides us with an opportunity to advance our understanding of its role in the pathophysiology of bladder dysfunction, and its potential mediation of the bladder cooling reflex. In this study, we report the distribution of the cool and menthol receptor TRPM8 in the urinary bladder in patients with overactive and painful bladder syndromes, and its relationship with clinical symptoms.

**Methods:**

Bladder specimens obtained from patients with painful bladder syndrome (PBS, n = 16), idiopathic detrusor overactivity (IDO, n = 14), and asymptomatic microscopic hematuria (controls, n = 17), were immunostained using specific antibodies to TRPM8; nerve fibre and urothelial immunostaining were analysed using fibre counts and computerized image analysis respectively. The results of immunohistochemistry were compared between the groups and correlated with the Pain, Frequency and Urgency scores.

**Results:**

TRPM8-immunoreactive staining was observed in the urothelium and nerve fibres scattered in the suburothelium. The nerve fibre staining was seen in fine-calibre axons and thick (myelinated) fibres. There was marked increase of TRPM8-immunoreactive nerve fibres in IDO (P = 0.0249) and PBS (P < 0.0001) specimens, compared with controls. A significantly higher number of TRPM8-immunoreactive axons were also seen in the IDO (P = 0.0246) and PBS (P < 0.0001) groups. Urothelial TRPM8 and TRPM8-immunoreactive thick myelinated fibres appeared unchanged in IDO and PBS. The relative density of TRPM8-immunoreactive nerve fibres significantly correlated with the Frequency (r = 0.5487, P = 0.0004) and Pain (r = 0.6582, P < 0.0001) scores, but not Urgency score.

**Conclusion:**

This study demonstrates increased TRPM8 in nerve fibres of overactive and painful bladders, and its relationship with clinical symptoms. TRPM8 may play a role in the symptomatology and pathophysiology of these disorders, and may provide an additional target for future overactive and painful bladder pharmacotherapy.

## Background

Despite considerable progress in understanding the patho-physiology of bladder dysfunction, there is presently no consistently effective treatment for disorders like the painful or overactive bladder syndromes. Painful bladder syndrome (PBS) is a chronic bladder hypersensitivity disorder that typically presents with suprapubic pain related to bladder filling, accompanied by other symptoms such as increased frequency and nocturia, in the absence of a definable aetiology [1]. The overactive bladder syndrome (OAB) is symptom complex characterized by urinary urgency with or without urge incontinence, usually with frequency and nocturia [2]. Detrusor overactivity is often the underlying condition. Detrusor overactivity should be further qualified as neurogenic detrusor overactivity (NDO), when there is a relevant neurologic condition or idiopathic detrusor overactivity (IDO), when there is no defined cause [2]. The recent discovery of a range of receptors in the bladder which respond to capsaicin, menthol, and temperature, and their expression in subsets of sensory nerve fibres, provides an opportunity to advance our understanding and treatment of these bladder disorders.

The mammalian sensory system is capable of detecting and discriminating thermal stimuli over a broad temperature spectrum. Within this range, temperatures over 43°C and below 15°C evoke not only a thermal sensation, but also a feeling of pain [3]. Six thermosensitive ion channels have been identified and cloned, all of which belong to the transient receptor potential (TRP) superfamily of cation channels [3,4]. These thermo-TRP channels exhibit distinct thermal activation thresholds [3,4], allowing us to sense and differentiate a large spectrum of temperatures, from below 0°C to 50°C. The physiological roles have yet to be determined for most members of this family, though their activation by specific chemical ligands and genetic evidence has clearly implicated certain TRP channels in the detection or transduction of a range of sensory stimuli [5].

The existence of bladder receptors sensitive to cold has been hypothesized since Bors and Blinn(1957) first reported a human bladder cooling reflex [6]. Experiments in cats showed that bladder thermosensation involves an association of cold sensitive receptors associated with unmyelinated C-fiber afferent neurons [7] and an intravesical infusion of a menthol solution increased the threshold temperature needed to trigger C-fibers, suggesting that these responses were likely mediated by a receptor sensitive to cold and menthol [8]. Subsequently, similar sensitization was noted in humans suggesting that these receptors also exist in the human bladder [9]. In 2002, a major breakthrough in the study of cold thermosensation was achieved, when two groups independently cloned and characterized this nonselective cation channel sensitive to cold temperatures and menthol, TRPM8 (also known as CMR1) [10,11]. It belongs to the 'long', or melastatin, subfamily of the transient receptor potential (TRP) family of ion channels and is activated by menthol, eucalyptol, icilin, and by temperatures below 25°C [12,13].

TRPM8 was initially identified as a prostate-specific TRP channel that was upregulated in malignant tissue [14]. Subsequent work detected TRPM8 in DRG and trigeminal ganglia neurons, where it has been shown to be involved in thermosensation [10,11]. Recently, TRPM8 has been identified in a number of human genitourinary tract tissues, including urinary bladder [15]. The reason for the existence of the cool and menthol receptor TRPM8 in the urinary tract is, however, still unknown. It has been proposed that the cold receptors in the urinary tract may have the same functional role as other thermoreceptors found elsewhere in the body, that participate in the regulation and maintenance of a stable central core temperature [16,17]. This is supported by the fact that body cooling is usually associated with an increased diuresis and thus the bladder cooling reflex has presumably evolved to help relieve the thermal ballast in the bladder when under cooling stress [16]. In a recent study, TRPM8 has been suggested to influence the cystometric parameters (micturition pressure and volume threshold for micturition) in guinea pigs [18]. This may have an effect on the voiding symptoms, such as frequency and urgency which are typical in bladder dysfunctions like the overactive and painful bladder syndromes.

To further our understanding of role of TRPM8 in the pathophysiology of bladder dysfunction, and discover any relationship with clinical symptoms, we have studied the expression of TRPM8 receptors in overactive and painful bladder syndromes.

## Methods

### Tissue specimens

Bladder tissue specimens was obtained from 17 control subjects under investigation for asymptomatic microscopic haematuria, 14 subjects with idiopathic detrusor overactivity and 16 subjects with painful bladder syndrome [19]. The mean (range) age of the controls was 51.1 years (21–89 years), IDO 52.2 years (32–73 years) and PBS patients 46.7 years (22–67 years). Approval by the local ethics committee (Reference No. – 00/5940 granted by Hammersmith, Queen Charlotte's and Chelsea and Acton Hospitals REC) and informed consent were obtained from patients and control subjects. Clinical assessments of these subjects included history and clinical examination, followed by midstream urine specimen (MSU), culture and cytology and urodynamics (CMG). The Severity of Symptoms assessment was carried out using the PUF Questionnaire [20]. In addition, the 'Pain score' was also recorded on a Visual analogue scale (VAS) on a scale of 0 to 10. All the controls had a 'pain score' of 0 (No Pain). In PBS patients, the 'pain score' ranged between 1 (mild pain) to 3 (severe pain) on the PUF scale and 3 to 8 on the visual analogue scale. The mean 'pain score' of PBS group was 2.5(PUF) and 5.9(VAS). The 'Frequency score' was obtained from PUF questionnaire and was rated as 0 (3 – 6 voids per day), 1 (7 – 10), 2 (11 – 14), 3 (15 – 19) and 4 (20 +). Similarly, 'Urgency score' obtained from PUF questionnaire and was graded as 0 (No urgency), 1 (Mild), 2 (Moderate) and 3 (Severe). All of the PBS patients complained of frequency (>5 in 12 h), nocturia (>2), urgency and suprapubic /pelvic pain without any signs of detrusor overactivity on urodynamics. The IDO patients presented with overactive bladder symptoms – urgency, with or without urge incontinence, frequency and nocturia and showed involuntary detrusor contractions during the filling phase of urodynamics [2].

Flexible or rigid cystoscopic bladder biopsies were obtained from a consistent site, just above and lateral to the ureteric orifices. A urine specimen was sent for culture before each cystoscopy. All patients had sterile urine cultures at the time of cystoscopy and biopsy.

Human tooth pulp specimens were immunostained using the same staining protocol as the bladder specimens.

### Preparation and staining of specimens

The samples were examined by histology (haematoxylin-eosin staining) and immunohistochemistry.

### Histology: Haematoxylin-eosin

Each specimen was immediately fixed in freshly prepared 4% paraformaldehyde w/v (PFA) in phosphate buffered saline for 60 minutes and then transferred to 0.45 M sucrose in phosphate buffered saline and refrigerated overnight. The samples were embedded in OCT medium and 10 μM sections were cut using a cryostat and stained with haematoxylin and eosin. The sections were studied for inflammatory changes, site of inflammation, vascularity and urothelial changes (hyperplasia and dysplasia).

### Antibodies

TRPM8: Affinity-purified rabbit antibody (GlaxoSmithKline, D SEL -2, Rabbit 1323) against cysteine tagged TRPM8 N-terminal peptide CEKWNYKKHTKEFPTDAFGD, corresponding to amino acids sequences 85–105 was used at a dilution of 1:1500. For specificity of TRPM8- immunostaining, the antibody was pre-incubated for 2 hours with decreasing concentration of peptide to TRPM8 before incubating with the tissue section.

### Immunohistochemistry

Frozen specimens were embedded in OCT medium and 30 μm frozen sections collected onto PLL (Poly-L-lysine; Sigma-Aldrich, Dorset, U.K.) coated slides. Sections were fixed in freshly prepared 4% w/v paraformaldehyde in phosphate-buffered saline (0.1 M phosphate; 0.9% w/v saline; pH 7.3). Endogenous peroxidase was blocked by incubation with 0.3% w/v hydrogen peroxide in methanol. After a wash in phosphate-buffered saline, the tissue sections were incubated with primary antibodies in dilutions mentioned above. Methodological controls included omission of primary antibodies, or their replacement with non-immune serum. Sites of antibody attachment were revealed using the nickel enhanced ABC (peroxidase) method [21]. Nuclei were counterstained with 0.1% w/v aqueous neutral red.

### Image analysis

TRPM8-immunoreactive nerve fibres were counted using a grid, as they were relatively fine and sparse. Grid fields were arranged to cover the whole tissue section, and the mean of two "blinded" observers, expressed as fibres per unit grid area (0.5 mm^2^), was used for analysis. Computerised image analysis using 'analySIS (version 5.0)' software was performed to semi-quantify urothelial staining. Images were captured via video link to an Olympus BX50 microscope (×40 objective) and scanned by the computer. Positive immunostaining was obtained after setting the grey level detection limits to threshold, and the area of highlighted fibres obtained as % area of the field scanned. Five fields per tissue section, chosen at random from a grid, were scanned and the mean value was used for analysis.

### Statistical analysis

Data was analysed between the groups by Mann Whitney U test using GraphPad Prism software, P < 0.05 indicated significance. The immunohistochemistry results were correlated with 'Pain, Frequency and Urgency scores' and analysed using Spearman's Correlation 'r'.

## Results

### Histopathology

Urothelium was present in all specimens. Histology was reported as normal in all controls, IDO and five PBS biopsies. The remaining eleven PBS bladder biopsies showed non-specific chronic inflammation with dispersed mast cells.

### Immunohistochemistry

**Figure 1:**
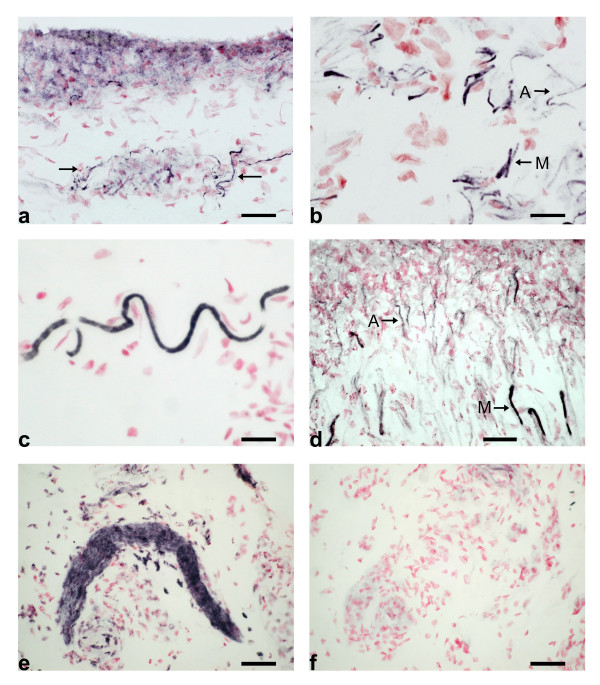
**TRPM8 immunoreactivity in human urinary bladder and dental pulp**. TRPM8 urothelial staining and suburothelial fibres (arrowed) in urinary bladder (a), Scale bar = 100 μm. TRPM8-immunoreactive axon (A)(b) and myelin (M)(b-c) staining in suburothelium, Scale bar = 50 μm. Similar, TRPM8-immunoreactive axon (A) and myelin (M) staining in dental pulp (d), Scale bar = 100 μm. TRPM8-immunoreactive nerve fibres in suburothelium (e) are abolished after pre-incubation with excess peptide with TRPM8 antibody at a dilution of 1:1500 (f), Scale bar = 100 μm. Nuclei counterstained with neutral red.

TRPM8-immunoreactive staining was observed in the urothelium and nerve fibres scattered in the suburothelium (Fig 1a). The TRPM8 antibody immunostained fine-calibre axons (presumably unmyelinated C-fibres) and both axonal and myelin staining of myelinated fibres (presumably A-delta fibres) (Fig 1b–c). A similar pattern of TRPM8 staining of fine axons and myelin was also seen in human tooth pulp (Fig 1d), using two different TRPM8 antibodies (the current GSK1323/SEL-2 and a commercially available antibody to TRPM8, H050-50 Phoenix Pharmac, USA), and the same staining protocol. Pre-incubation of the TRPM8 antibody with an excess of peptide to TRPM8 completely abolished the urothelial and nerve fibre TRPM8-immunoreactivity (Fig 1e–f). The immunostaining gradually reappeared with decreasing concentration of the peptide, supporting the specificity of the TRPM8-immunostaining.

**Figure 2:**
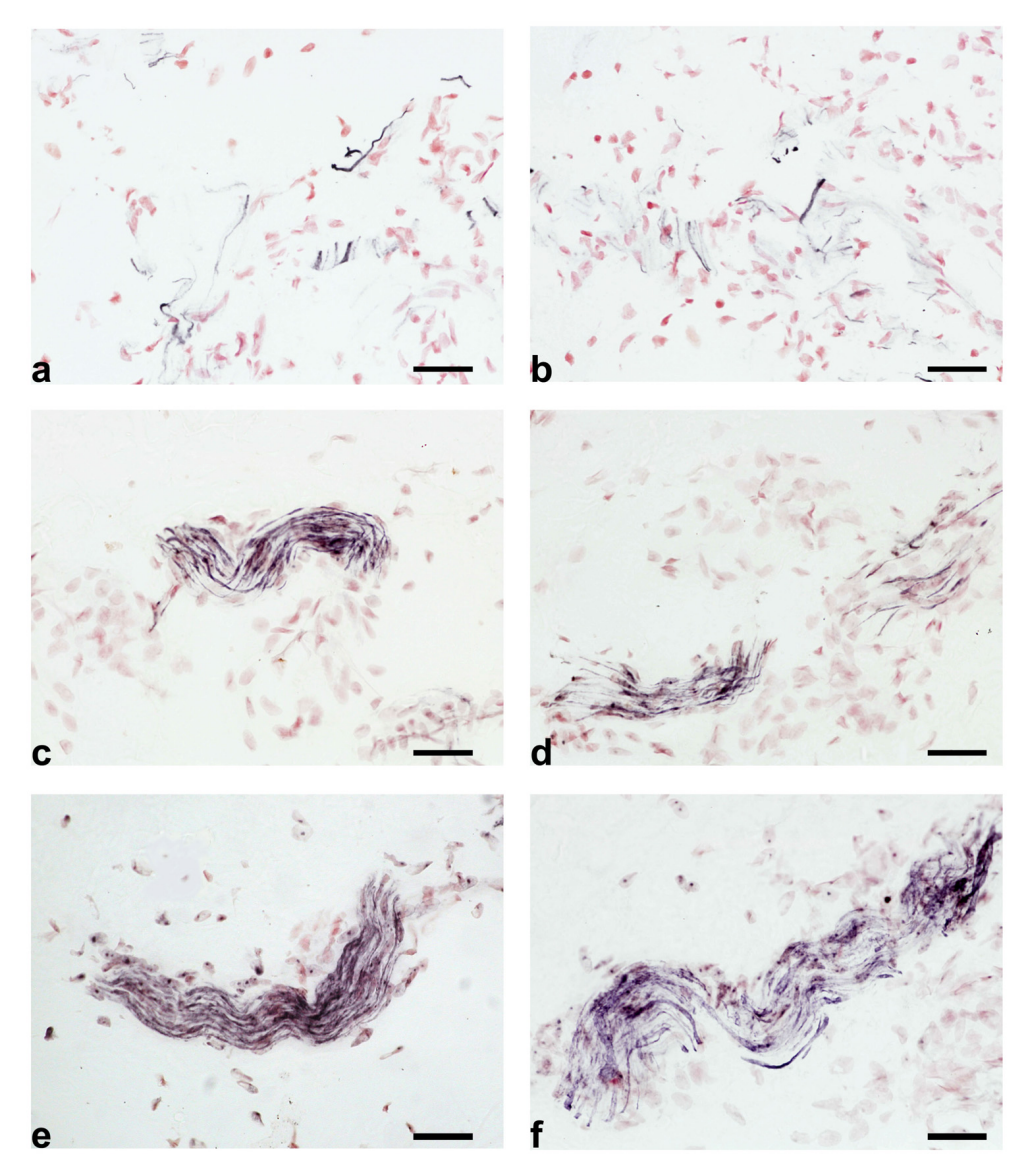
**TRPM8-immunoreactive staining of nerve fibres in suburothelium**. TRPM8-immunoreactive staining of nerve fibres in suburothelium of control (a-b), IDO (c-d) and PBS (e-f) bladders. Nuclei counterstained with neutral red. Scale bar = 100 μm.

**Figure 3:**
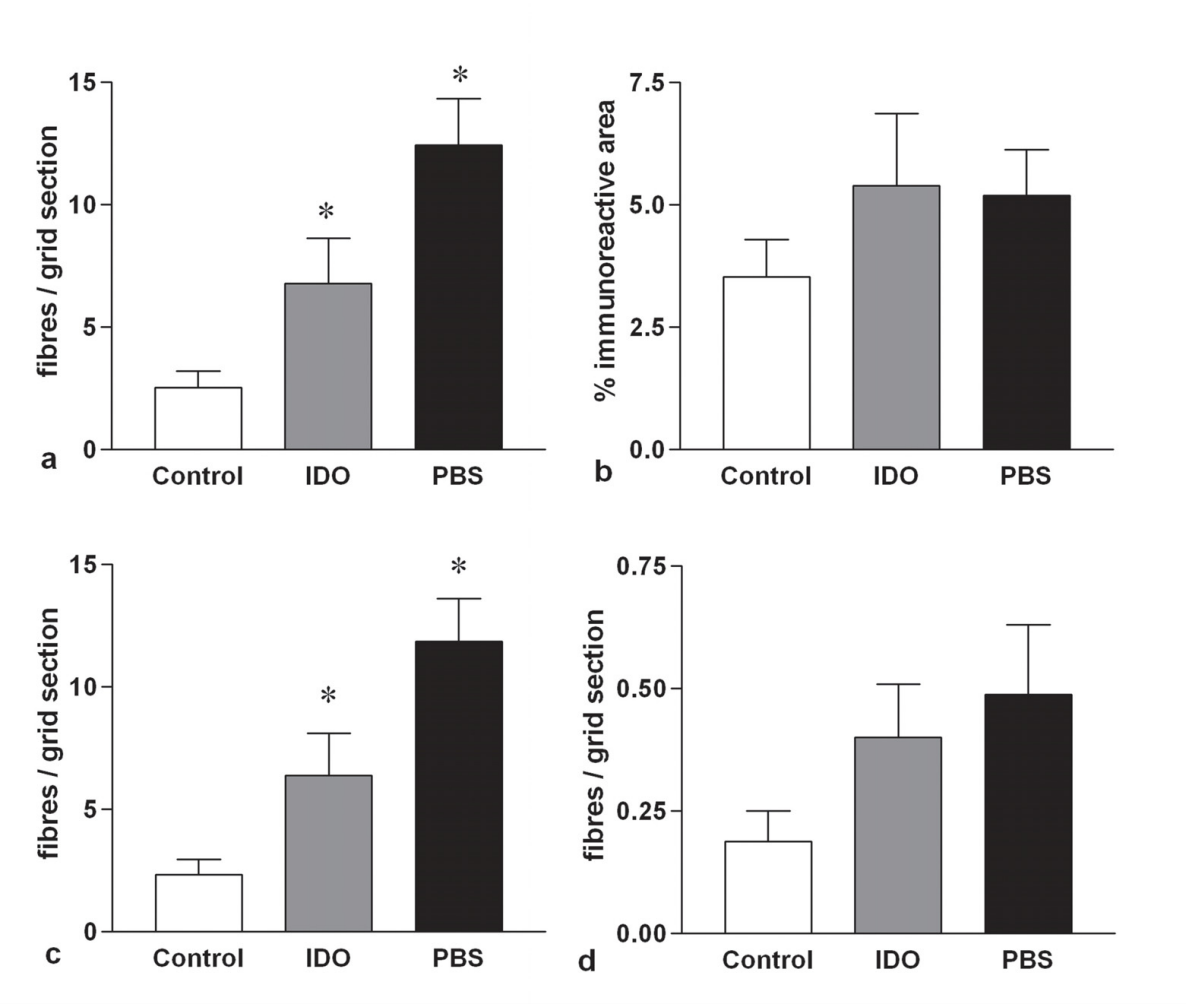
**Image analysis of TRPM8 immunostaining in control and bladder disorders**. Bar charts showing the relative numbers of TRPM8-immunoreactive total nerve fibres(a), axons(c) and myelinated fibres(d) per unit grid area (Mean ± SEM) and relative % area (Mean ± SEM) of TRPM8 urothelial immunoreactivity(b) in control (n = 17), IDO (n = 14) and painful bladder syndrome(n = 16) groups.

TRPM8-immunoreactive nerve fibres were seen scattered throughout the suburothelium of the control (Fig 2a–b), IDO (Fig 2c–d) and PBS (Fig 2e–f) bladder specimens. Compared with control, the IDO (P = 0.0249) and PBS (P < 0.0001) bladder specimens had a significantly larger number of fibres immunoreactive to TRPM8 (Fig 3a). On separately analysing the TRPM8-immunoreactive fine-calibre axons, a significant increase, similar to total nerve fibres was seen in the IDO (P = 0.0246) and PBS (P < 0.0001) groups (Fig 3c). Compared to controls, a three- and five-fold increase of TRPM8-immunoreactive axons was seen in IDO and PBS groups respectively. The myelin stained fibres also showed a two-fold increase in the PBS and IDO groups, but this was not statistically significant (PBS, P = 0.143, IDO, P = 0.076; Fig 3d).

There was no statistically significant difference in the TRPM8 urothelial immunostaining between controls and IDO (P = 0.1555) or PBS (P = 0.1816) groups (Fig 3b).

### Correlation with clinical scores

**Figure 4:**
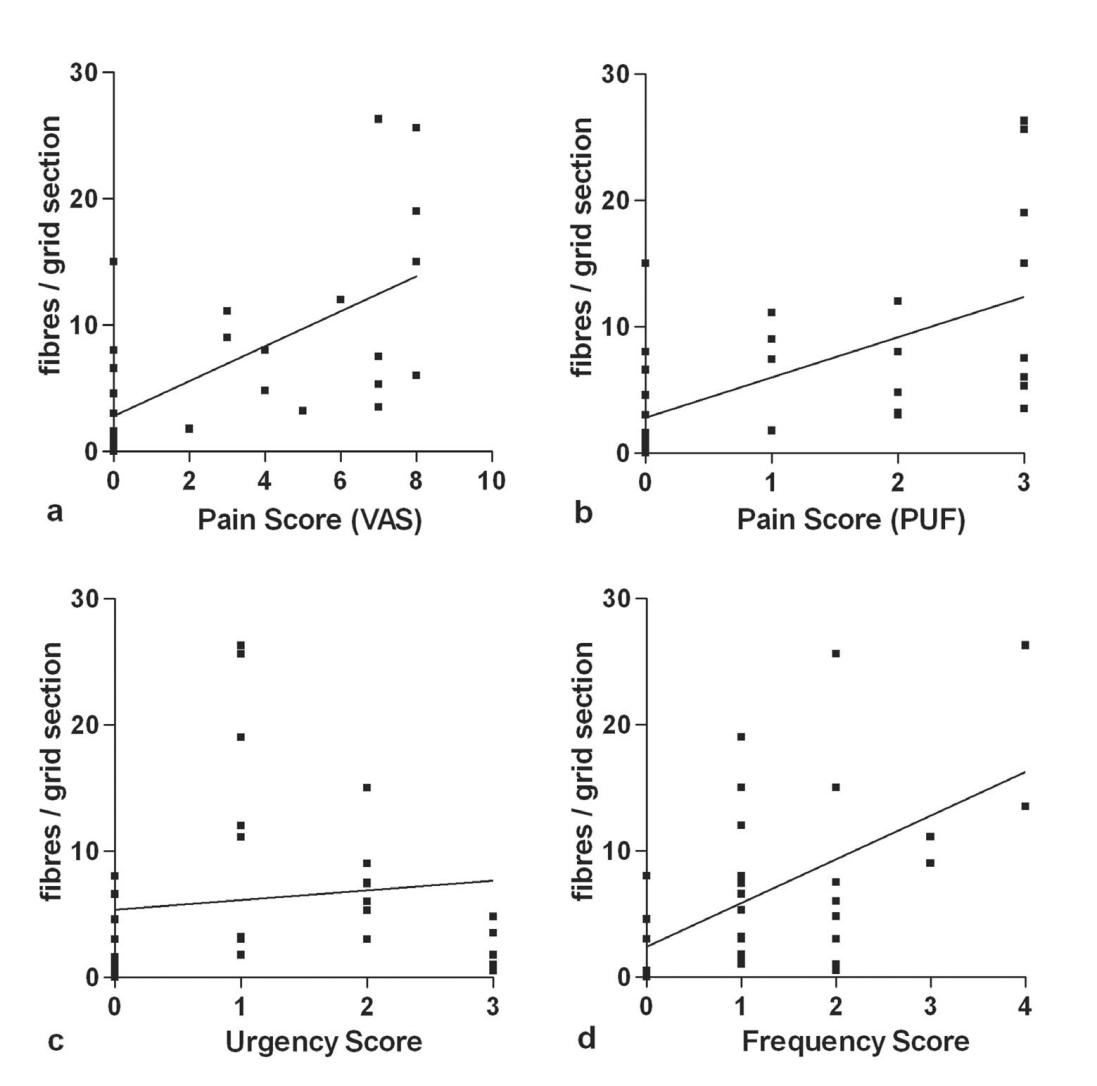
**Correlation of TRPM8-immunoreactive nerve fibres with clinical scores**. Scatter charts showing the correlation of TRPM8-immunoreactive total nerve fibres with (a) Pain Score (VAS; r = 0.6582, P < 0.0001); (b) Pain Score (PUF; r = 0.6165, P < 0.0001); (c) Urgency Score (r = 0.2997, P = 0.0715) and (d) Frequency Score (r = 0.5487, P = 0.0004) in all bladder specimen groups (n = 37).

The relative density of TRPM8-immunoreactive nerve fibres significantly correlated with the Pain Score (VAS) (r = 0.6582, P < 0.0001; Fig 4a). Similar correlation was seen with 'Pain scores' measured via the PUF scale (r = 0.6165, P < 0.0001) (Fig 4b). No correlation was found, however, between TRPM8 urothelial immunoreactivity and Pain score (VAS, r = 0.0713, P = 0.6978, PUF, r = 0.05448, P = 0.7596; Fig not shown).

No correlation was found between the 'Urgency score' and the TRPM8-immunoreactive nerve fibre count (r = 0.2997, P = 0.0715; Fig 4c) or TRPM8 urothelial immunoreactivity (r = 0.1661, P = 0.3478; Fig not shown). The 'Frequency score' significantly correlated with the TRPM8-immunoreactive nerve fibre count (r = 0.5487, P = 0.0004; Fig 4d), but not with TRPM8 urothelial immunoreactivity (r = 0.0438, P = 0.8053; Fig not shown).

**Figure 5:**
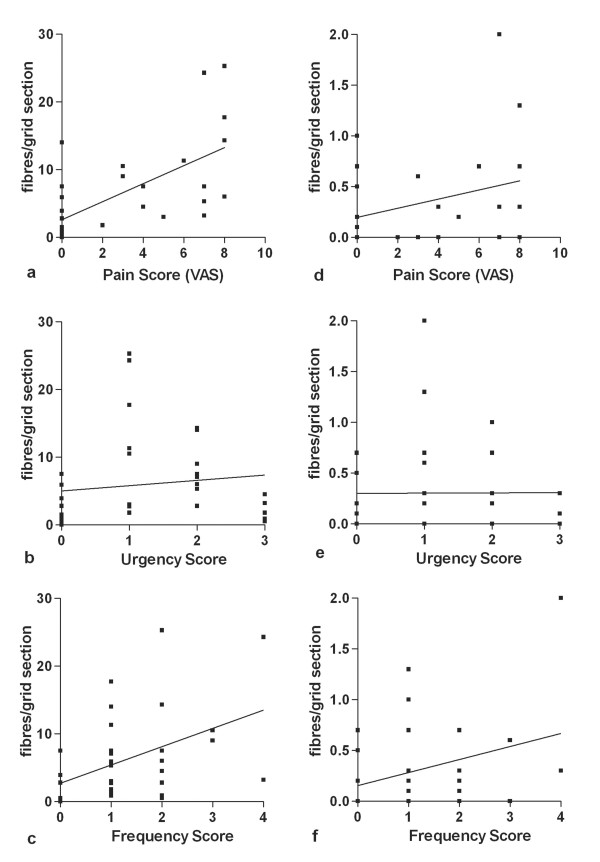
**Correlation of TRPM8-immunoreactive axons and myelin fibres with clinical scores**. Scatter charts showing the correlation of TRPM8-immunoreactive axon fibres with (a) Pain (VAS; r = 0.6783, P < 0.0001); (b) Urgency (r = 0.3102, P = 0.0617); (c) Frequency Scores (r = 0.5057, P = 0.0014) and the correlation of TRPM8-immunoreactive myelin fibres with (d) Pain (VAS; r = 0.2321, P = 0.1866); (e) Urgency (r = 0.09731, P = 0.5667); (f) Frequency (r = 0.2179, P = 0.1951) scores in all bladder specimen groups (n = 37).

Similar results were seen when the TRPM8-immunoreactive axons were correlated with Pain (VAS, r = 0.6783, P < 0.0001, PUF, r = 0.6258, P < 0.0001), Urgency (r = 0.3102, P = 0.0617) and Frequency (r = 0.5057, P = 0.0014) scores (Fig 5a–c). However, the correlations with TRPM8-immunoreactive myelinated fibres were not significant for any these scores (Pain, VAS, r = 0.2321, P = 0.1866, PUF, r = 0.2119, P = 0.2080; Urgency, r = 0.09731, P = 0.5667; Frequency, r = 0.2179, P = 0.1951) (Fig 5d–f), which might be due to the relative paucity of these myelinated fibres in the specimens.

## Discussion

In this study, we have demonstrated the cellular locations of cool and menthol receptor TRPM8 in the human urinary bladder. The presence of TRPM8 in the bladder has been reported in previous studies [15]. Stein et al recently demonstrated the presence of TRPM8-mRNA in the human urinary bladder using RT-PCR, and by immunofluorescence, TRPM8 in rat bladder and human urothelial cells in culture [15]. While they showed that TRPM8 was localized within the bladder urothelial layer, in our study TRPM8 immunoreactive fibres were seen scattered in the suburothelium and nerve bundles, in addition to urothelial staining. The different findings in these studies may reflect the affinity and/or avidity of the antibodies, and the methods used. Lindstrom and Mazieres [8], and Geirsson et. al [9] performed studies suggesting the presence of cold and menthol sensitive receptors associated with unmyelinated C-fibres in the cat and human bladders. TRPM8 has been shown to be predominantly expressed in small-diameter neurons in sacral DRG in guinea pig, while the staining in large diameter cells was diffuse or absent [18]. Abe and colleagues [22] have reported the distribution of TRPM8 in the trigeminal ganglion. They observed TRPM8 immunoreactivity in a subset of neurons with diameter of cell bodies in the range of 22.1 ± 4 μm that can be classified as small neurons (origin of C or A delta fibres). They further characterized the TRPM8-positive neurons by double staining analysis with anti-NF200, a marker of A delta fibres, and anti-TRPM8 antibodies. TRPM8-immunoreactivity was found in a subset of both NF200-negative and -positive neurons, and it was concluded that TRPM8 was expressed in cell bodies of A delta and C fibres. In agreement with Abe et al, TRPM8 appeared to stain both axons (possibly unmyelinated, C fibres) and myelin (possibly myelinated, A delta fibres) in our study, with two different antibodies. A similar pattern of TRPM8 staining of fine axons and myelin was also seen in human tooth pulp. However, this differentiation of myelinated and unmyelinated fibres needs to be confirmed by further studies with selective markers. This data must be interpreted with caution, even though we have established TRPM8 specificity with conventional methods – we may have in these TRPM8 antibodies, at the very least, a marker of both unmyelinated and myelinated fibres.

In control bladders, the TRPM8 immunoreactive fibres were fine and few in number. The TRPM8-immunoreactive staining was also detected in nerve fibre bundles, but was extremely scarce. In contrast, in IDO and PBS bladders, TRPM8-immunoreactive fibres were seen in nerve bundles along with fine fibres scattered in the suburothelium. The presence of increased immunoreactivity in nerve bundles may indicate increased levels of axonally transported TRPM8 in IDO and PBS bladders.

In the present study, we show that TRPM8-immunoreactive nerve fibres were significantly increased in painful bladder syndrome, and correlated with pain score. While the role of TRPM8 in cold signaling is well known, its role in nociception is debatable. Menthol, when applied at low concentrations to the skin or mouth, elicits a pleasant cool sensation, while higher doses can cause burning, irritation and pain through activation and sensitization of C-fibers [23]. Identification of endogenous ligands for TRPM8 would provide further clues as to the pathophysiological functions of this channel.

A salient finding of the study was a five-fold increase of TRPM8-immunoreactive axons (ummyelinated fibres) in PBS as compared to controls. The micturition reflex is normally mediated by small myelinated A-delta afferents which respond to bladder distension; the C-fibres are inactive and are therefore termed "silent C-fibres" [24]. However, they may become hyperactive during inflammation, exhibiting spontaneous firing when the bladder is empty, and increased firing during bladder distension [24]. In chronic inflammatory bladder diseases (e.g. in interstitial cystitis/PBS), hyperexcitability of C-fibre afferent pathways has been proposed as a mechanism for urgency and bladder pain [25]. This hypothesis is supported in our study by the correlation of these fibres with pain. While there was no significant difference in urothelial TRPM8 immunostaining in PBS and IDO specimens or its correlation with clinical scores, further studies using different methods of quantification, turnover and function of urothelial TRPM8 receptors are necessary before it is concluded that they do not play a role in the pathophysiology of IDO or PBS.

The regulators of TRPM8 expression in the bladder are not known. In a previous study, we have demonstrated increased expression of TRPV1 fibres in PBS [26]. TRPM8 does not appear to be co-expressed with TRPV1 [11,27]. However, in DRG cultures, which have Nerve Growth Factor(NGF)-rich conditions, capsaicin and menthol sensitivity are observed functionally in many neurons [10,27]. It may be hypothesized that in PBS patients, the high NGF levels [28,29] lead to a phenotypic change in sensory fibres which normally express TRPV1 alone, but which now also express TRPM8 [30], resulting in a similar increase of both receptors. Alternatively, visceral (pelvic) afferents may be polymodal, and normally express both TRPV1 and TRPM8, with increases of both receptors in pathological states [31]. This can be supported by the fact that capsaicin prevents the cold response in guinea-pig bladders pre-treated with menthol [18], suggesting that TRPM8 might be expressed in the C-afferent neurons, which also express TRPV1. Further studies are needed of rodent and human bladder pelvic afferents that originate in sacral DRG neurons to address these alternatives.

In a recent study, Tsukimi and colleagues [18] demonstrated that intravesical menthol administration increased the micturition pressure and decreased the volume threshold for micturition. It has been proposed that menthol decreases the threshold for C-fiber activation by enhancing spontaneous activity. Therefore, it is possible that TRPM8 may play a role in the regulation of threshold for initiating micturition reflex. This proposal is supported by our finding of significant correlation of frequency score with the TRPM8-immunoreactive nerve fibres. In addition, an increase in TRPM8-immunoreactive nerve fibres in IDO and PBS, suggests that TRPM8 may contribute to the increased frequency which is a prominent symptom in these conditions. Further correlations with clinical studies, such as the ice water test (to elicit the bladder cooling reflex in both PBS and detrusor overactivity) and more accurate methods of clinical measurement, including voiding diaries and urodynamic results, are in progress to determine the functional consequences of the TRPM8 receptor increase.

## Conclusion

This study shows a significant increase of TRPM8-immunoreactive nerve fibres in suburothelium from patients with overactive and painful bladder syndrome. A significant correlation was also seen between the relative density of the TRPM8-immunoreactive nerve fibres with the pain and urinary frequency. Based on these findings we propose that the role and therapeutic potential of TRPM8 deserves further study, and may provide an additional target for future overactive and painful bladder pharmacotherapy.

## List of abbreviations

PBS : Painful bladder syndrome

IDO : Idiopathic detrusor overactivity

TRPM8 : Transient receptor potential melastatin – 8

TRPV1 : Transient receptor potential vanilloid – 1

DRG : Dorsal root ganglion

VAS : Visual analogue scale

PUF Scale : Pain, Urgency and Frequency Scale

NGF : Nerve Growth Factor

SEM : Standard error of the Mean

RT-PCR : Reverse Transcriptase Polymerase Chain Reaction

OCT : Optimal cutting temperature compound

## Competing interests

The author(s) declare that they have no competing interests.

## Authors' contributions

GM was involved in collection of specimens, clinical assessment of patients, analysis of data and writing the manuscript. YY performed the immunohistochemistry, analysis of data and helped writing the manuscript. GDS, ISS, and SLC were responsible for the design and production of the TRPM8 antibodies used, CDB and CB helped conceive the study, with interpretation of the data, and writing the manuscript. SKA performed the surgical procedures and participated in the conception of the study. PA conceived the study and participated in its design and coordination, interpretation and completion of the manuscript. All authors read and approved the manuscript

## Pre-publication history

The pre-publication history for this paper can be accessed here:
